# Prognostic value of microRNA-21 in intra- and extrahepatic cholangiocarcinoma after radical resection: cohort study

**DOI:** 10.1093/bjsopen/zrae031

**Published:** 2024-04-18

**Authors:** Lenka N C Boyd, Mahsoem Ali, Annalisa Comandatore, Giovanni Brandi, Simona Tavolari, Raffaele Gaeta, Laura L Meijer, Tessa Y S Le Large, Mattia Riefolo, Francesco Vasuri, Luca Morelli, Hanneke W M van Laarhoven, Elisa Giovannetti, Geert Kazemier, Ingrid Garajová

**Affiliations:** Department of Surgery, Amsterdam UMC, Location Vrije Universiteit, Amsterdam, The Netherlands; Department of Medical Oncology, Lab of Medical Oncology, Amsterdam UMC, Location Vrije Universiteit, Amsterdam, The Netherlands; Cancer Center Amsterdam, Imaging and Biomarkers, Amsterdam, The Netherlands; Department of Surgery, Amsterdam UMC, Location Vrije Universiteit, Amsterdam, The Netherlands; Department of Medical Oncology, Lab of Medical Oncology, Amsterdam UMC, Location Vrije Universiteit, Amsterdam, The Netherlands; Cancer Center Amsterdam, Imaging and Biomarkers, Amsterdam, The Netherlands; Department of Surgery, Amsterdam UMC, Location Vrije Universiteit, Amsterdam, The Netherlands; Department of Medical Oncology, Lab of Medical Oncology, Amsterdam UMC, Location Vrije Universiteit, Amsterdam, The Netherlands; General Surgery Unit, Department of Translational Research and New Technologies in Medicine and Surgery, University of Pisa, Pisa, Italy; Center for Applied Biomedical Research, Sant'Orsola-Malpighi University Hospital, Bologna, Italy; Center for Applied Biomedical Research, Sant'Orsola-Malpighi University Hospital, Bologna, Italy; Second Division of Surgical Pathology, University Hospital of Pisa, Pisa, Italy; Department of Surgery, Amsterdam UMC, Location Vrije Universiteit, Amsterdam, The Netherlands; Department of Surgery, Amsterdam UMC, Location Vrije Universiteit, Amsterdam, The Netherlands; Cancer Center Amsterdam, Imaging and Biomarkers, Amsterdam, The Netherlands; Pathology Unit, IRCCS Azienda Ospedaliero-Universitaria di Bologna, Bologna, Italy; Pathology Unit, IRCCS Azienda Ospedaliero-Universitaria di Bologna, Bologna, Italy; General Surgery Unit, Department of Translational Research and New Technologies in Medicine and Surgery, University of Pisa, Pisa, Italy; Department of Medical Oncology, Lab of Medical Oncology, Amsterdam UMC, Location Vrije Universiteit, Amsterdam, The Netherlands; Department of Medical Oncology, Amsterdam UMC, Location University of Amsterdam, Amsterdam, The Netherlands; Department of Medical Oncology, Lab of Medical Oncology, Amsterdam UMC, Location Vrije Universiteit, Amsterdam, The Netherlands; Cancer Center Amsterdam, Imaging and Biomarkers, Amsterdam, The Netherlands; Cancer Pharmacology Lab, Fondazione Pisa per la Scienza, Pisa, Italy; Department of Surgery, Amsterdam UMC, Location Vrije Universiteit, Amsterdam, The Netherlands; Cancer Center Amsterdam, Imaging and Biomarkers, Amsterdam, The Netherlands; Department of Medical Oncology, Lab of Medical Oncology, Amsterdam UMC, Location Vrije Universiteit, Amsterdam, The Netherlands; Medical Oncology Unit, University Hospital of Parma, Parma, Italy

## Introduction

The clinical outcomes of patients with cholangiocarcinoma are poor, with a median survival of 17–38 months after resection, depending on the anatomical location of the primary tumour and the presence of high-risk features (for example positive resection margins and lymph node metastases)^1,2^.

Incorporating biomarkers such as microRNAs into prognostic models for cholangiocarcinoma might improve patient risk assessment, enable personalized clinical decision-making and provide more precise prognosis estimates. A previous meta-analysis identified microRNA-21 (miR-21) as an ‘ideal prognostic marker for clinical decision-making’ but it did not compare its performance with commonly used clinicopathological variables such as resection margin and tumour grade. Previous studies revealed that the prognostic value of a novel biomarker can be substantially reduced after correcting for commonly measured prognostic variables^3–5^.

The aim of this study was to assess the association between miRNA-21 and overall survival (OS) in cholangiocarcinoma and assess its performance in prognostic prediction models.

## Methods

### Study cohort

Data from patients who underwent curative-intent resection for cholangiocarcinoma between July 2002 and July 2021 at the University of Bologna, University of Pisa, and Amsterdam University Medical Center were extracted from electronic medical records. Adjuvant chemotherapy with gemcitabine was administered to all patients from the University of Bologna and Pisa, but not to those from the Amsterdam University Medical Center. The study was approved by the institutional review board of each participating centre.

### RNA extraction and quantitative real-time PCR

Details regarding the RNA extraction, expression analyses and data normalization are provided in the *Supplementary materials*. Total RNA was isolated from 10 μm thick formalin-fixed paraffin-embedded tissue (FFPE) tumour sections. RNA was used for expression analysis of miR-21 by quantitative real-time PCR (qPCR). RNA (10–100 ng) was reverse transcribed and the resulting cDNA was amplified using the specific Taqman MicroRNA assays (Life Technologies) for miR-21 and RNU6B (assay ID, 000397 and 001093 respectively).

### Statistical analysis

Continuous and categorical baseline variables were reported as median (interquartile range) or as numbers and percentages respectively. Cox regression modelling was used to assess the association between miR-21 and OS, and to assess the added value of miR-21 to routinely available prognostic markers in a multivariable model. In the multivariable model, the following prespecified prognostic covariates were included: miR-21, age, sex, lymphatic invasion, vascular invasion, perineural invasion, resection margin, T stage and N stage. Age and miR-21 were modelled using restricted cubic splines^6^.

Missing data were handled using multiple imputation (60 imputations)^7^. The imputation model included the event variable, the Nelson–Aalen estimate of the cumulative baseline hazard and all prognostic covariates included in the full Cox regression model^8^.

A *P* value lower than 0.05 was considered statistically significant. All statistical analyses were performed in R, version 4.2.1 (R Foundation for Statistical Computing), and Stata, version 17.0 (StataCorp). Details regarding the statistical analyses are provided in the *Supplementary materials*.

## Ethics approval and consent to participate

All patients gave informed consent. The protocol was approved by the University of Bologna Ethics Committee. All methods were carried out in accordance with relevant guidelines and regulations.

## Results

### Baseline characteristics

A total of 131 extrahepatic and intrahepatic cholangiocarcinoma patients aged between 34 and 82 years were included. Baseline characteristics are shown in *Table 1* and the frequency of missing data is shown per variable in *Table S1*.

**Table 1:** Baseline characteristics

Variable	Extrahepatic cholangiocarcinoma (N = 103)	Intrahepatic cholangiocarcinoma (N = 26)
Age (years), median (i.q.r.)	65 (50–72)	61 (57–72)
**Sex**		
Female	47 (46)	12 (46)
Male	56 (54)	14 (54)
Vascular invasion	22 of 74 (30)	2 of 20 (10)
Lymphatic invasion	64 of 94 (68)	7 of 20 (35)
**T stage**		
T1	15 (17)	1 (5)
T2	44 (50)	10 (45)
T3	29 (33)	11 (50)
**N stage**		
N0	35 (36)	10 (56)
N1	62 (64)	8 (44)
miR-21, cycle threshold, median (i.q.r.)	24 (23–25)	24 (23–25)
Survival (years), median (i.q.r.)	2.0 (1.0–7.5)	6.3 (3.8–6.3)

Values are *n* (%) unless otherwise indicated. i.q.r., interquartile range; miR-21, microRNA-21.

### OS stratified by miR-21 expression

**Fig. 1:**
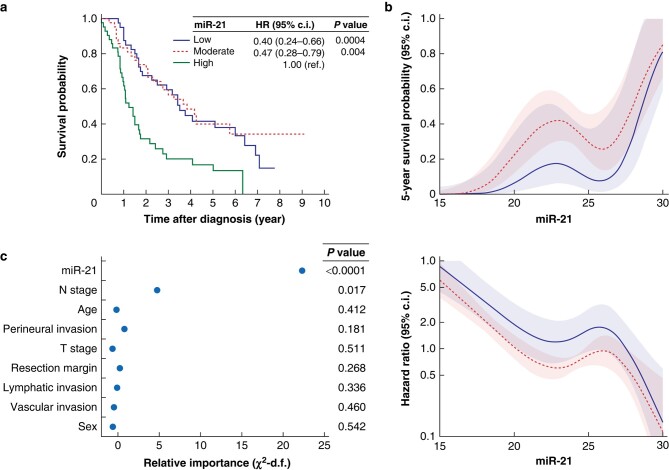
**Prognostic value of miRNA-21 in cholangiocarcinoma**. **a**, Kaplan–Meier curve for three equally sized groups of patients with low, moderate and high expression of miRNA-21; **b**, adjusted (blue) and unadjusted (black) association between miR-21 and overall survival; **c**, importance of each prognostic variable in the full Cox regression model, as measured by the partial Wald χ^2^ minus the predictor degrees of freedom. Higher χ^2^ values indicate higher prognostic value. **a**, low miR-21, Ct cycle 24.6 to 30 (9 ICC, 34 ECC); moderate miR-21, Ct cycle 22.9 to 24.6 (9 ICC, 35 ECC); high miR-21, Ct cycle 15 to 22.9 (8 ICC, 34 ECC); **b**, miR-21, number of Ct cycles for miR-21; **c**, Wald χ^2^ values are derived from the fully adjusted Cox regression model, after multiple imputation. ICC, intrahepatic cholangiocarcinoma; ECC, extrahepatic cholangiocarcinoma; Ct, cycle threshold; miR-21, microRNA-21.

OS in three equally sized groups of patients with low, moderate and high miRNA-21 expression is shown in *Fig. 1a*. Compared with the group of patients with high miR-21 expression, moderate miR-21 expression and low miR-21 expression were associated with significantly longer OS (hazard ratio (HR) 0.47 (95% c.i. 0.28 to 0.79) and 0.49 (0.24 to 0.66) respectively). This association was similar between men and women (interaction test, *P* = 0.422), and remained consistent across age groups (interaction test, *P* = 0.255).

### Association between miR-21 and OS

In univariable analysis, miR-21 was strongly associated with shorter OS (standardized HR per standard deviation increase in miR-21 2.13 (95% c.i. 1.56 to 2.94); *P* < 0.0001). The association between miR-21 and OS remained consistent (adjusted standardized HR 2.08 (95% c.i. 1.54 to 2.86); *P* < 0.0001; *Fig. 1b*), after correcting for conventional clinicopathological variables (that is age, sex, vascular invasion, perineural invasion, lymphatic invasion, resection margin, T stage and N stage). This association was similar between patients with intrahepatic *versus* extrahepatic cholangiocarcinoma and remained consistent in four sensitivity analyses (*Supplementary results*).

### Incremental prognostic value of miR-21

In the fully adjusted Cox regression model, the prognostic value of miR-21 was higher than all conventional clinicopathological variables (*Fig. 1c*), and miR-21 had significant prognostic value after correcting for these variables (likelihood ratio test, *P* < 0.0001). In the fully adjusted model, miR-21 contained 70% of the prognostic information provided by all clinical, pathological and biomarker variables combined (likelihood ratio χ^2^ before and after adding miR-21, 21.4 *versus* 66.7). Prognostic performance increased substantially when adding miR-21 to a Cox regression model including all conventional clinicopathological variables (Harrell’s *C-*statistic, 0.76 *versus* 0.66; difference in *C-*statistic, 0.09 (95% c.i. 0.04 to 0.14); *P* = 0.0002). The incremental prognostic value of miR-21 remained similar after correcting for overfitting (difference in overoptimism-corrected *C*-statistic, 0.10).

## Discussion

In this cohort study of 131 patients with intrahepatic or extrahepatic cholangiocarcinoma, miR-21 expression was strongly and independently associated with OS allowing patient stratification into distinct prognostic risk groups and adding prognostic value over conventional clinicopathological variables. The association between miR-21 and OS was consistent in direction and magnitude between men and women, across age groups and across several sensitivity analyses.

In a recent meta-analysis, miR-21 has been described as a potential prognostic biomarker for cholangiocarcinoma. The methodology of the current analysis strengthens these results^9–13^. In contrast to previous analyses, miR-21 was not dichotomized at an arbitrary cut off (for example the median)^7,14,15^; the multiple imputation approach used avoided discarding patients with missing data, and allowed a higher statistical accuracy; the study was not focused on the crude unadjusted association between high *versus* low miR-21 expression and OS but miR-21 performance was adjusted for known prognostic covariates.

Previous studies have shown that a novel marker may not be an independent (prognostic) marker, after correction for routinely measured biomarkers and clinicopathological variables^3,16–19^. In contrast, a substantial increase in prognostic performance was observed in this study after adding miR-21 into a prediction model with known prognostic factors (increase in *C*-statistic, 0.09), validating miR-21 as a strong independent prognostic factor in cholangiocarcinoma.

Limitations of this study included its retrospective nature and the heterogeneity in adjuvant chemotherapy use across centres. Some prognostic covariates were not considered in multivariable analyses (for example WHO performance status, carbohydrate antigen 19-9 and bilirubin) which could attenuate the association between miR-21 and OS. Future studies could assess whether the prognostic value of miR-21 decreases after correcting for these additional prognostic variables.

This study validated miR-21 as a strong, independent predictor of OS in cholangiocarcinoma. Subject to further validation, the addition of miR-21 to established prognostic models for cholangiocarcinoma may improve patient risk assessment and facilitate more personalized clinical decision-making after surgery. Validation studies should assess the added (clinical) value of miR-21 across different subgroups with sufficient statistical precision^20,21^. Prospective clinical studies should assess whether the use of miR-21 could improve clinical decision-making after surgery.

## Supplementary Material

zrae031_Supplementary_Data

## Data Availability

Data sets generated and analysed during the present study are available from the corresponding author upon reasonable request.
