# Supplementary material for: Prognostic value of microRNA-21 in intra- and extrahepatic cholangiocarcinoma after radical resection: cohort study
Source: BJS Open. 2024 Apr 18;8(2):zrae031. doi: 10.1093/bjsopen/zrae031 (PMC11026099; doi:10.1093/bjsopen/zrae031)
Supplement: zrae031_Supplementary_Data [file zrae031_supplementary_data.docx]

**Prognostic value of microRNA-21 in intra- and extra-hepatic cholangiocarcinoma**

**after radical resection: cohort study**

Lenka N.C. Boyd^1,2,3,†^, Mahsoem Ali^1,2,3,†^, Annalisa Comandatore^1,2,4^, Giovanni Brandi^5^, Simona Tavolari^5^, Raffaele Gaeta^6^, Laura L. Meijer^1^, Tessa Y.S. Le Large^1,3^, Mattia Riefolo^7^, Francesco Vasuri^7^, Luca Morelli^4^, Hanneke W.M. van Laarhoven^2,8^, Elisa Giovannetti^2,3,9^, Geert Kazemier^1,3††^, Ingrid Garajová^2,10††^

^1^ Amsterdam UMC, Location Vrije Universiteit, Department of Surgery, Amsterdam, the Netherlands

^2^ Amsterdam UMC, Location Vrije Universiteit, Department of Medical Oncology, Lab of Medical Oncology, Amsterdam, the Netherlands

^3^ Cancer Center Amsterdam, Imaging and Biomarkers, Amsterdam, the Netherlands

^4^ General Surgery Unit, Department of Translational Research and New Technologies in Medicine and Surgery,

University of Pisa, Pisa, Italy

^5^ Center for Applied Biomedical Research, Sant'Orsola-Malpighi University Hospital, Bologna, Italy

^6^ Second Division of Surgical Pathology, University Hospital of Pisa, Italy.

^7^ Pathology Unit, IRCCS Azienda Ospedaliero-Universitaria di Bologna, Bologna, Italy

^8^ Amsterdam UMC, Location University of Amsterdam, Department of Medical Oncology, Amsterdam,

the Netherlands

^9^ Cancer Pharmacology Lab, Fondazione Pisa per la Scienza, Pisa, Italy

^10^ Medical Oncology Unit, University Hospital of Parma, Parma, Italy

^†^ Shared first authorship

^††^ Shared last authorship

**Corresponding author**: Dr. Elisa Giovannetti, Dept. Medical Oncology, VU University Medical Center, Cancer Center Amsterdam; De Boelelaan 1117, 1081 HV Amsterdam, The Netherlands; and Cancer Pharmacology Lab, Fondazione Pisa per la Scienza, Pisa, Italy; E-mail: [elisa.giovannetti@gmail.com](mailto:elisa.giovannetti@gmail.com); Twitter: @elisagiovan; ORCID ID: 0000-0002-7565-7504; Tel: +31204442267.

**Supplementary Materials - Index**

| **Supplementary Methods** | *pag. 3* |
| --- | --- |
| **Supplementary Results** | *pag. 6* |
| **Supplementary Figures and Tables** | *pag. 7* |
| **References** | *pag. 8* |
|  |  |

**Supplementary Methods**

*RNA extraction from FFPE tissues*

Total RNA was isolated from 10 μm thick formalin-fixed paraffin-embedded tissue (FFPE) tumour sections using the RecoverAll^TM^ Total Nucleic Acid Isolation Kit for FFPE from Ambion (Austin, TX, USA) (#AM1975) according to the manufacturer’s instructions. The tumour cell content was evaluated by experienced pathologists. Sample quality was assessed by Nanodrop (ND-1000, Isogen Life Science, The Netherlands). The extracted RNA was eluted in 60μl of RNase free H_2_O. The samples were either stored at −80°C or directly processed further.

*Reverse transcription and quantitative real-time PCR*

RNA was used for expression analysis of miR-21 by quantitative Real-Time PCR (qPCR). RNA (10–100 ng) was reverse transcribed and the resulting cDNA was amplified using the specific Taqman MicroRNA assays (Life Technologies) for miR-21 and RNU6B (assay ID, 000397 and 001093, respectively). The PCRs were performed in the 7500HT sequence detection system (Applied Biosystems), in accordance with the manufacturer’s instructions.

*Data normalization*

All assays were performed in duplicate and results which did not meet methodological quality control criteria were omitted. Amplification data were normalized to RNU6B expression. Quantification of relative microRNA expression was performed using the 2^(-ΔCt) method.^1,2^

*Statistical analysis*

Continuous and categorical baseline variables were reported as median (interquartile range) or as numbers and percentages, respectively.

Cox proportional hazards regression was used to assess the association between miRNA-21 and overall survival, and to assess the added value of miRNA-21 to routinely available prognostic markers in a multivariable model. In the multivariable model, the following prognostic covariates were included: miRNA-21, age, sex, lymphatic invasion, vascular invasion, perineural invasion, resection margin, T stage, and N stage. To assess whether the association between miR-21 and overall was different across age groups and between men and women, a likelihood ratio test for the interaction term was used. All variables were pre-specified based on subject matter expertise and well-established prognostic factors in cholangiocarcinoma. Variable selection procedures (eg, backward selection or forward selection) were not performed to reduce the degree of overfitting.^3^ Overall survival stratified by miR-21 expression (low, moderate, and high) was visualized using Kaplan-Meier curves, by creating three equally sized groups with miR-21 expression at or below the 33^rd^ percentile, between the 34^th^ and 67^th^ percentile, and higher than the 67^th^ percentile.

The functional form of continuous variables and the proportional hazards assumption in the Cox regression model was examined using visual inspection of Martingale residuals and Schoenfeld residuals, respectively. The Grambsch-Therneau test was used to formally test potential violation of the proportional hazards assumption; however, there was no statistical evidence for nonproportional hazards (global Grambsch-Therneau test, P=0.35). Age and miRNA-21 were modelled using restricted cubic splines with three and four knots, respectively, after winsorization of miRNA-21 at the 5^th^ and 95^th^ percentile to reduce the influence of extreme values.^4^ Collinearity was assessed using variance inflation factors.

Missing data were handled using multiple imputation with additive regression, predictive mean matching, and bootstrapping under the missing at random assumption.^3^ The imputation model included the event variable (i.e., whether a patient had died or was censored), the Nelson-Aalen estimate of the cumulative baseline hazard, and all prognostic covariates (i.e., miRNA-21, age, sex, vascular invasion, lymphatic invasion, perineural invasion, resection margin, T stage, and N stage). The event variable and the Nelson-Aalen estimate of the cumulative baseline hazard were used in the imputation model, as this approach is recommended for multiple imputation with time-to-event data.^5^ Age was modelled in the imputation model as a restricted cubic spline with three knots.^3^ In total, 60 imputations and 100 bootstrap resamples were used.^6^ Model parameter estimates were pooled across imputed datasets using Rubin’s rules.

Four sensitivity analyses were performed to assess the robustness of our results. ^7^ First, Firth’s correction (ie, penalized partial maximum likelihood estimation) was used to account for potential sparse data bias in the Cox regression model. Second, multivariate imputation by chained equations (60 imputations and 20 iterations) and complete case analysis were used as alternative approaches to handle missing data. Third, continuous variables were modelled using multivariable fractional polynomials instead of restricted cubic splines. Lastly, stratification for centre was used in the Cox regression model to correct for potential differences between centres.

A P value lower than 0.05 was considered to indicate statistical significance. All statistical analyses were performed in R, version 4.2.1 (R Foundation for Statistical Computing), and Stata, version 17.0 (StataCorp).

**Supplementary Results**

In the low, moderate, and high miR-21 expression group, the 5-year restricted mean survival time (RMST) was respectively 3.4 years (95% CI, 2.8 to 3.9 years), 3.1 years (95% CI, 2.5 to 3.6 years), and 2.1 years (95% CI, 1.6 to 2.6 years). Compared to the high miR-21 group, overall survival was significantly prolonged in the moderate miR-21 group (difference in 5-year RMST, 12 months; 95% CI, 3 to 21 months; P=0.011) and the low miR-21 group (difference in 5-year RMST, 15 months; 95% CI, 7 to 24 months; P=0.001).

*Sensitivity analyses*

Four sensitivity analyses were performed. First, the association between miR-21 and overall survival remained similar after Firth’s correction for potential sparse data bias (adjusted standardized HR, 2.22; 95% CI, 1.75 to 2.86; P<0.0001). Second, the hazard ratio of miR-21 was attenuated when using multivariate imputation by chained equations to handle missing data (adjusted standardized HR, 1.61; 95% CI, 1.18 to 2.16; P=0.004); in contrast, the prognostic value of miR-21 was larger in a complete case analysis (adjusted standardized HR, 2.78; 95% CI, 1.79 to 4.17; P<0.0001). Third, model estimates were similar in a multivariable fractional polynomial model (adjusted standardized HR, 2.27; 95% CI, 1.75 to 2.94; P<0.0001), although there was no evidence of a nonlinear relationship between miR-21 and overall survival when modelling miR-21 as a fractional polynomial term. Lastly, the hazard ratio of miR-21 was 1.85 (95% CI, 1.20 to 2.86; P=0.005), when stratifying for centre.

**Supplementary Figures and Tables**

| **Variable** | **Extrahepatic cholangiocarcinoma (N=103)** | | **Intrahepatic cholangiocarcinoma (N=26)** | |
| --- | --- | --- | --- | --- |
| Age, n (%) | 0 |  | 0 |  |
| Sex, n (%) | 0 |  | 0 |  |
| Vascular invasion, n/N (%) | 29 | (28%) | 6 | (23%) |
| Lymphatic invasion, n/N (%) | 9 | (9%) | 6 | (23%) |
| T stage, n (%) | 15 | (15%) | 4 | (15%) |
| N stage, n (%) | 6 | (6%) | 8 | (31%) |
| miR-21, cycle threshold – median (IQR) | 43 | (42%) | 0 |  |

**Table S1. Missing data per variable.** Number and percentage of patients with missing data for each variable, stratified by the type of cholangiocarcinoma.

**References**

1. Schmittgen TD, Jiang J, Liu Q, Yang L. A high‐throughput method to monitor the expression of microRNA precursors. *Nucleic acids research* 2004; **32**(4): e43-e.

2. Shi R, Chiang VL. Facile means for quantifying microRNA expression by real-time PCR. *Biotechniques* 2005; **39**(4): 519-25.

3. Harrell FE. Regression modeling strategies: with applications to linear models, logistic regression, and survival analysis: Springer; 2001.

4. Steyerberg EW, Steyerberg EW. Coding of categorical and continuous predictors. *Clinical prediction models: A practical approach to development, validation, and updating* 2019: 175-90.

5. White IR, Royston P. Imputing missing covariate values for the Cox model. *Statistics in medicine* 2009; **28**(15): 1982-98.

6. Austin PC, White IR, Lee DS, van Buuren S. Missing data in clinical research: a tutorial on multiple imputation. *Canadian Journal of Cardiology* 2021; **37**(9): 1322-31.

7. Van Buuren S. Flexible imputation of missing data: CRC press; 2018.
